# Supplementary material for: A long noncoding RNA acts as a post-transcriptional regulator of heat shock protein (HSP70) synthesis in the cold hardy Diamesa tonsa under heat shock
Source: PLoS One. 2020 Apr 2;15(4):e0227172. doi: 10.1371/journal.pone.0227172 (PMC7117718; doi:10.1371/journal.pone.0227172)
Supplement: S1 Fig — (A). Nucleotide and deduced amino acid sequence of hsc70-I. (B) Phylogenetic tree inferred from nucleotide sequences of hsp70 in different dipteran species. (C) Phylogenetic tree inferred from the inferred amino-acid sequence of HSP70 in different dipteran species. (DOCX) [file pone.0227172.s001.docx]

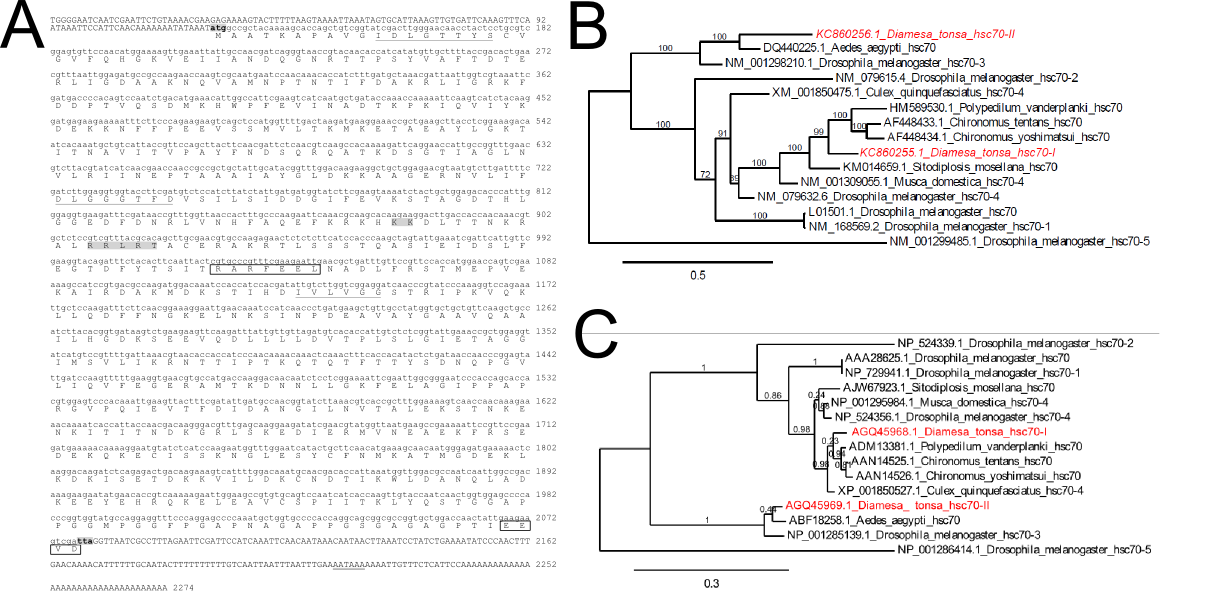


**S1 Fig.** (**A**). Nucleotide and deduced amino acid sequence of *hsc70-I* from *Diamesa tonsa*. In the nucleotide sequence, upper case indicates 5ʹUTR and 3ʹUTRs and lower case indicates the coding region. The start (ATG) and stop codon (TTA) are shadowed and in bold, and the consensus polyA signal in the 3ʹUTR is in italic with a double-underline. The three characteristic HSP70 family signatures are underlined, and the non-organellar consensus motif (RARFEEL) and the cytoplasmic C-terminal region EEVD are shown in a box. The putative bipartite nuclear localization signal (KK and RRLRT) is shadowed in grey. (**B**) Phylogenetic tree inferred from nucleotide sequences of hsp70 in different dipteran species. The tree was constructed using Phylogeny.fr tool at ExPASy Proteomics server (http://www.phylogeny.fr) using the “One Click” mode with default settings. The numbers above the branches are tree supported values generated by PhyML using the approximate Likelihood-Ratio (aLRT) statistical test. (**C**) Phylogenetic tree inferred from the inferred amino-acid sequence of HSP70 in different dipteran species. The tree was constructed using Phylogeny.fr tool at ExPASy Proteomics server (http://www.phylogeny.fr) using the “One Click” mode with default settings. The numbers above the branches are tree supported values generated by PhyML using the approximate Likelihood-Ratio (aLRT) statistical test.
