# Supplementary material for: A long noncoding RNA acts as a post-transcriptional regulator of heat shock protein (HSP70) synthesis in the cold hardy Diamesa tonsa under heat shock
Source: PLoS One. 2020 Apr 2;15(4):e0227172. doi: 10.1371/journal.pone.0227172 (PMC7117718; doi:10.1371/journal.pone.0227172)
Supplement: S2 Fig — (DOCX) [file pone.0227172.s002.docx]

GCGGCTTTAGCAATCATAAGTGTCTCAGCCAGTGAAGACAAAAAAGAAATTGGAACAGTCATTGGAATTGATTTAGGTACCACGTATTCA 90

TGTGTTGGAGTGTACAAAAACGGTCGCGTAGAAATTATTGCCAACGATCAAGGTAACAGAATTACTCCATCATATGTGGCGTTCACAGCT 180

GAAGGAGAGCGTCTTATTGGAGATGCTGCTAAGAATCAACTTACAACAAATCCTGAAAATACCGTTTTCGATGCTAAACGTTTGATCGGT 270

CGTGAATGGAGTGACGCAGCAGTGCAACACGACATCAAATTCTTCCCCTTCAAGGTTCTCGAGAAGAATACCAAACCACACATTGAAGTC 360

AAGACAAGCCAAGGTAACAAGGTGTTTGCACCTGAAGAAATTTCAGCT**atg**gttcttttgaaaatgaaggaaaccgctgaagcttatctc 450

M V L L K M K E T A E A Y L

ggaaagaaggtcacacacgctgtcgtcacagttccagcttatttcaacgatgcccaacgtcaagcaacaaaagatgctggagttattgct 540

G K K V T H A V V T V P A Y F N D A Q R Q A T K D A G V I A

ggattaaatgtcatgagaattatcaacgaaccaacagccgcagctattgcatacggtttagacaagaaggatggagagaaaaatgtttta 630

G L N V M R I I N E P T A A A I A Y G L D K K D G E K N V L

gtctttgatttgggtggtggtaccttcgatgtctcacttttgaccattgataacggagtctttgaagttgttgcaacaaacggtgacaca 720

V F D L G G G T F D V S L L T I D N G V F E V V A T N G D T

catttgggtggtgaagatttcgatcaacgtgttatggatcatttcattaaattgtacaagaagaagaagggcaaggatatcagaaaagac 810

H L G G E D F D Q R V M D H F I K L Y K K K K G K D I R K D

aaccgtgctgtccaaaaattacgtcgtgaagttgaaaaggctaaacgtgctctttctgccagtcagcaagttcgcattgaaattgaatca 900

N R A V Q K L R R E V E K A K R A L S A S Q Q V R I E I E S

ttctttgaaggtgaagacttctccgaatccttatctcgtgctaagttcgaagaattgaacatggatttgttccgttccacattgaaaccc 990

F F E G E D F S E S L S R A K F E E L N M D L F R S T L K P

gtccaaaaagtattggaagatgctgatatgaacaaaaaagatgtcgatgaaattgttttggtcggaggatcaacacgtattccaaaagta 1080

V Q K V L E D A D M N K K D V D E I V L V G G S T R I P K V

caacaactcgttaaagaattcttcaacggaaaggaaccatcacgtggaatcaatcctgatgaagctgtcgcttatggtgctgctgttcaa 1170

Q Q L V K E F F N G K E P S R G I N P D E A V A Y G A A V Q

gctggagtcttatctggtgaacaagatactgatgccatcgtcttgttggatgttaatcctttgaccatgggtattgaaaccgttggtgga 1260

A G V L S G E Q D T D A I V L L D V N P L T M G I E T V G G

gtcatgacaaaattgatcccaagaaacaccgttattccaacaaagaaatcacaaattttctcaactgcttccgataaccaacacacagtc 1350

V M T K L I P R N T V I P T K K S Q I F S T A S D N Q H T V

acaattcaagtttatgaaggtgaacgtccaatgaccaaagataaccatttattaggaaaattcgacttgactggaattccaccagcacca 1440

T I Q V Y E G E R P M T K D N H L L G K F D L T G I P P A P

agaggtattccacaaattgaagtctcattcgaaattgacgccaacggtattttgcaagtgtcagctgaagacaagggtacaggaaatcgc 1530

R G I P Q I E V S F E I D A N G I L Q V S A E D K G T G N R

gaaaagattgttattaccaatgaccaaaatcgtttgacacctgatgacatcgaacgtatgattaaggacgctgaacgttttgctgatgat 1620

E K I V I T N D Q N R L T P D D I E R M I K D A E R F A D D

gacaagaaactgaaggaacgcgttgaagccagaaatgaattggaaagttatgcatacagtcttaagaaccaattgggagacaaagaaaag 1710

D K K L K E R V E A R N E L E S Y A Y S L K N Q L G D K E K

ttaggagcaaaattatctgatgaagaaaaatcaaagatggaagaagcaattgatgagaagatcaagtggctcgaggaaaaccaagacaca 1800

L G A K L S D E E K S K M E E A I D E K I K W L E E N Q D T

gactctgaagaatacagaaaacaaaagaaagaattagaagaaattgttcaaccaatcattgctaaactctacgctggaacaggtggagta 1890

D S E E Y R K Q K K E L E E I V Q P I I A K L Y A G T G G V

ccaccaactggcggcgaagaggaagacgaagatctcaaggatgagttg**taa**ATGTTCATTTCATCTTAGTCGAAGTTTCTTTAGTTCTTT 1980

P P T G G E E E D E D L K D E L * TTTTTTATTTAATTTGTACAATTTTTTCGCTTAAGAGATAGTACTTTATTGTCGTCGACTCTTCCCTACCTTCCACAAAACACACACACA 2070

CACAAAAAACCCCATGTCCCCCATTGTGTTTAACACACAAACACATGAAAAAAAAA

**S2** **Fig.** Sequence of the *Dt-hsc70-II* mRNA constitutive isoform.
