# Supplementary material for: A long noncoding RNA acts as a post-transcriptional regulator of heat shock protein (HSP70) synthesis in the cold hardy Diamesa tonsa under heat shock
Source: PLoS One. 2020 Apr 2;15(4):e0227172. doi: 10.1371/journal.pone.0227172 (PMC7117718; doi:10.1371/journal.pone.0227172)
Supplement: S3 Fig — (DOCX) [file pone.0227172.s003.docx]

**Hsp70** -----------------------------------------------------AAGCAGTGGTATCAACG 17

**Hsp70 intron** -TTTTTCATTTCCATGGCAGCTGAGAATATTGTAGGAGATCTTCTAGAAAGATAAGCAGTGGTATCAACG 69

*****************

**Hsp70** CAGAGTACATGGGGATTCATACAAGAAACGTAAAGCAACACACAAGTGAAA-TAAAACAAAGTTAAAGAC 86

**Hsp70 intron** CAGAGTACATGGGGATTCATACAAGAAACGTAAAGCAACACACAAGTGAAAATAAAACAAAGTTAAAGAC 139

*************************************************** ******************

**Hsp70** AAATTCAAAATAAAACTATTTAAGAAGTGACATAATTGTGCAGAAATAAAATATTGTGAATTAAATAAAA 156

**Hsp70 intron** AAATTCTAAATAAAACTATTGAAGAAGTGACATAATTGTGCAGAAATAAAATATTGTAAATTAAATAAAA 209

****** ************* ************************************ ************

**Hsp70** TATCTGAAAGTTAAATAAATCAAAGAATATTAAAAATGCCTTCCGCAACAAATAAAGTGGCTATTGGTAT 226

**Hsp70 intron** TATCTGAAAGTTAAATAAATCAAAGAATATTAAAAATGCCTTCCGCAACAAATAAAGTGGCTATTGGTAT 279

**********************************************************************

**Hsp70** CGACTTGGGAACAACATATTCCTGCGTTGGTGTTTATCAACATGGAAAAGTGGAAATTATTGCTAATGAA 296

**Hsp70 intron** CGACTTGGGTACGACATACTCATGTGTTGGAGTTTATCAACATGGAAAAGTGGAAATTATTGCTAATGAA 349

********* ** ***** ** ** ***** ***************************************

**Hsp70** AATGGAAACAGAACAACACCCAGCTATGTAGCATTTAATGATACAGAAAGATTAATTGGAGATGCTGCCA 366

**Hsp70 intron** ATGGGAAACAGAACAACACCCAGCTATGTAGCATTTAATGATACAGAAAGATTAATTGGAGATGCTGCCA 419

* *******************************************************************

**Hsp70** AAAATCAA-------------------------------------------------------------- 374

**Hsp70 intron** AAAATCAG*gtaagacatttatttttagatccactttgaacaatatttcaaaatacgtgacacaaattgct* 489

*******

**Hsp70** ---------------------------------------------------------------------- 374

**Hsp70 intron** *ttatggactaaactgtcctttattggaaagattatctgaatgaatccactgaatttataattagaatttt* 559

**Hsp70** ---------------------------------------------------------------------- 374

**Hsp70 intron** *caatggagacgcccaagaataatcatccaaagcatatctcaataacattattataacttatatttgcgtg* 629

**Hsp70** ---------------------------------------------------------------------- 374

**Hsp70 intron** *ggtattaaattactaacatcttattctttcttagagtgcgaatgatatagaaaaaccccgagatactttt* 699

**Hsp70** ---------------------------------------------------------------------- 374

**Hsp70 intron** *tatttaataatttctttttatttttgaagaaaacaagaaagaagtagaaaaatgtgttctattaataatt* 769

**Hsp70** ---------------------------------------------------------------------- 374

**Hsp70 intron** *ttgaaataattggaggagggaaatatttttaaatgtgaattttatgcatttaatttatttgttttatttg* 839

**Hsp70** ----------------GTGGCAATGAATCCTACTAACAGTGTCTTTGATGCTAAACGAATGATTGGACGC 428

**Hsp70 intron** *ttttctttcacttca*GGTTGCGATGAATCCGATAAATAGTGTTTTTGATGCAAAGCGGCTGATTGGACGT 909

** ** ******** * ** ***** ******** ** ** **********

**Hsp70** AAATTTGATGATGAAAAATTACAAGCTGATATGAAACATTGGCCATTCAAAGTCACCAATGATTGTGGAA 498

**Hsp70 intron** AAATTTGATGATGATAAAGTGCAATCTGACATGAAACATTGGCCATTCAAAGTCATCAATGATTGTGGAA 979

************** *** * *** **** ************************* **************

**Hsp70** AACCAAAGATCCAAGTTGAATTCAAAGGGGAAACCAAGACCTTTGCTCCAGAAGAAGTCAGTTCAATGAT 568

**Hsp70 intron** AACCAAAGATTCAAGTTGAATTCAAAGGGGAAACCAAAAGATTTGCACCAGAAGAAGTCAGTTCAATGAT 1049

********** ************************** * ***** ***********************

**Hsp70** TCTCACTAAGATGCGTGAAACTGCTGAAGTATATTTGGGACAAAAGGTTACTGATGCTGTTATTACTGTA 638

**Hsp70 intron** TCTTACGAAGATGCGTGAAACTGCTGAAGTATATTTGGGACAAAAGGTCACTGATGCTGTTATTACTGTA 1119

*** ** ***************************************** *********************

**Hsp70** CCAGCTTATTTCAATGATTCACAACGTCAAGCAACCAAAGATGCTGGAGCAATTGCTGGATTAAACGTTT 708

**Hsp70 intron** CCAGCTTATTTCAATGATTCACAGAGACAAGCAACCAAAGATGCTGGAGCAATTGCTGGATTAAATGTTT 1189

*********************** * ************************************** ****

**Hsp70** TGAGAATCATCAATGAACCTACTGCAGCTACATTGGCTTATGGTTTGGACAAGAACTTGAAAGGAGAAAA 778

**Hsp70 intron** TGAGAATTATCAATGAACCAACTGCTGCTGCATTAGCTTATGGCTTGGGCAAAAAAAAAAAAAAAAAAAA 1259

******* *********** ***** *** **** ******** **** *** ** *** * ****

**Hsp70** GAATGTTTTGATCTTTGATCTTGGTGGTGGAACTTTTGATGTTTCAATTCTTGCTATTGATCAAGGATCA 848

**Hsp70 intron** AA-------------------------------------------------------------------- 1261

*

**Hsp70** TTATTTGAAGTTAAATCGACTGCTGGTGACACACATTTGGGTGGTGAAGATTTTGATAATCGTTTGGTGA 918

**Hsp70 intron** ---------------------------------------------------------------------- 1261

**Hsp70** ATCATTTTGCTGAAGAATTCAAGAGAAAGTTCAAGAAAGACATGTCAGGAAATGCAAGAGCATTGCGTCG 988

**Hsp70 intron** ---------------------------------------------------------------------- 1261

**Hsp70** TTTAAGAACAGCCTGTGAACGTGCAAAGAGAACATTATCATCCAGTACTGAAGCCACTATTGAAATTGAT 1058

**Hsp70 intron** ---------------------------------------------------------------------- 1261

**Hsp70** GCATTACATGAAGGTGTTGACTTCAACTCAAAGATCTCTCGTGCTCGGTTTGAAGAAATGAACATGGATT 1128

**Hsp70 intron** ---------------------------------------------------------------------- 1261

**Hsp70** TATTCAGATCAACATTGGAACCAGTAGAACGTGCATTGAAAGACGCAAAGATGGACAAAGGAGCTGTACA 1198

**Hsp70 intron** ---------------------------------------------------------------------- 1261

**Hsp70** TGATGTTGTTCTTGTTGGTGGCTCAACTCGTATTCCAAAGATTCAAAAAATGCTTCAAGACTTCTTTGGA 1268

**Hsp70 intron** ---------------------------------------------------------------------- 1261

**Hsp70** GGAAAGACATTGAATCTTTCAATCAATCCTGATGAAGCTGTTGCATATGGAGCTGCTGTACAAGCTGCTA 1338

**Hsp70 intron** ---------------------------------------------------------------------- 1261

**Hsp70** TTCTTACTGGCGACACCAGTTCTACAATTCAGGATGTTTTGTTGGTTGATGTCACTCCACTATCATTGGG 1408

**Hsp70 intron** ---------------------------------------------------------------------- 1261

**Hsp70** TATTGAAACTGCTGGTGGTGTAATGACCAAATTGGTTGAACGCAATTCTAGAATTCCATGCAAGCAACAA 1478

**Hsp70 intron** ---------------------------------------------------------------------- 1261

**Hsp70** CAAACATTTACAACATACAGTGATAACCAGAATGCTGTTACCATCACTGTATTTGAAGGTGAACGAGCAA 1548

**Hsp70 intron** ---------------------------------------------------------------------- 1261

**Hsp70** TGGTCAAAGATAACAACTTGTTGGGAACATTTAATCTCACCGGAATTCCACCAGCACCACGTGGAGTACC 1618

**Hsp70 intron** ---------------------------------------------------------------------- 1261

**Hsp70** CAAAATTGAAGTTACATTTGATTTGAATGCTGATGGTATCCTCAATGTATCAGCCAAAGATAATTCAACT 1688

**Hsp70 intron** ---------------------------------------------------------------------- 1261

**Hsp70** GGAAAACAAGAAAAAATTACAATTAAAAACGACAAAGGACGTTTATCCAAAGCCGACATTGACCGAATGT 1758

**Hsp70 intron** ---------------------------------------------------------------------- 1261

**Hsp70** TGAGTGAAGCAGAAAAATATCGTGAAGAAGATGAGAAACAACAACAACGCATTGTAGCCAGAAATCAATT 1828

**Hsp70 intron** ---------------------------------------------------------------------- 1261

**Hsp70** GGAAAGTTACATTTTTGGATGCAAACAAGCTGCTGAAGATGCACCAGCAAACAAACTTACAGATGCTGAC 1898

**Hsp70 intron** ---------------------------------------------------------------------- 1261

**Hsp70** AAGAAAAGTGTCAAAGATAAATGCACCTCAGAACTTTCTTGGTTGGATACAAATACATTAGCTGAAAAAG 1968

**Hsp70 intron** ---------------------------------------------------------------------- 1261

**Hsp70** AAGAATTCGACGATCATCTTAAGGATGTACAAAAAGTGTGTGGTCCAATAATGGCAAAACTTCATGGAGC 2038

**Hsp70 intron** ---------------------------------------------------------------------- 1261

**Hsp70** TCAGCAAAATGGTGGTGCTCAACCAGCTGGAAAACCAACTGTTGAAGAAGTCGACTAATTTTATACTTAA 2108

**Hsp70 intron** ---------------------------------------------------------------------- 1261

**Hsp70** CTCATCATTGCATTTATCATTCATTAAACATTTATTATTTATTTCATAACCCCTTAAATGTTCATTTAGT 2178

**Hsp70 intron** ---------------------------------------------------------------------- 1261

**Hsp70** TTTAAAAAATAAGAAGTTGTTGAATAAATTACTAAAAAAAAAAAAAAAAAAAAAAAAAAAAAAAAA 2244

**Hsp70 intron** ------------------------------------------------------------------ 1261

**S3 Fig**. Alignment of the *Dt-hsp70* and the pseudogene transcripts.
