# Supplementary material for: A long noncoding RNA acts as a post-transcriptional regulator of heat shock protein (HSP70) synthesis in the cold hardy Diamesa tonsa under heat shock
Source: PLoS One. 2020 Apr 2;15(4):e0227172. doi: 10.1371/journal.pone.0227172 (PMC7117718; doi:10.1371/journal.pone.0227172)
Supplement: S4 Fig — (A) Correlation between fold change at translational and transcriptional level. (B) Schematic representation of the two hsp70 transcripts. (DOCX) [file pone.0227172.s004.docx]

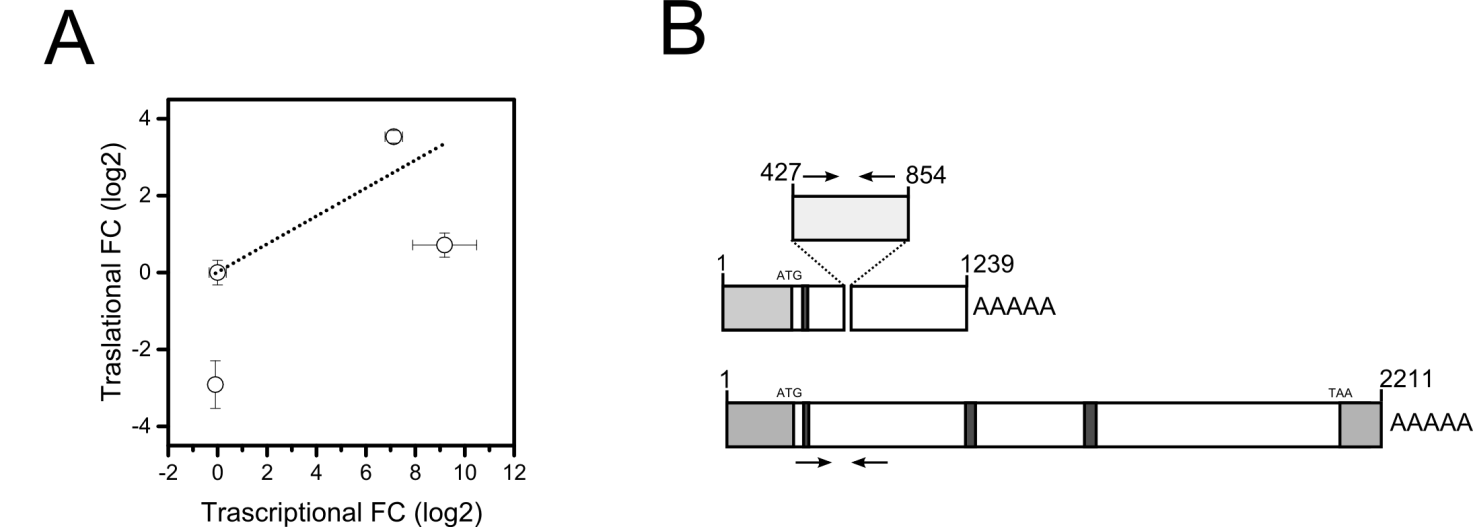


**S4 Fig**. (**A**) Correlation between fold change at translational and transcriptional level was calculated (R^2^=0.389). (**B**) Schematic representation of the two *hsp70* transcripts: light grey boxes are the 5ʹ and 3ʹ UTR, dark grey boxes indicated the position of the three characteristic HSP70 family domains. Arrows indicate the position of the Forward (F) and Reverse (R) primers used for further analysis.
