# Supplementary material for: A long noncoding RNA acts as a post-transcriptional regulator of heat shock protein (HSP70) synthesis in the cold hardy Diamesa tonsa under heat shock
Source: PLoS One. 2020 Apr 2;15(4):e0227172. doi: 10.1371/journal.pone.0227172 (PMC7117718; doi:10.1371/journal.pone.0227172)
Supplement: S1 Raw Images — (DOCX) [file pone.0227172.s005.docx]

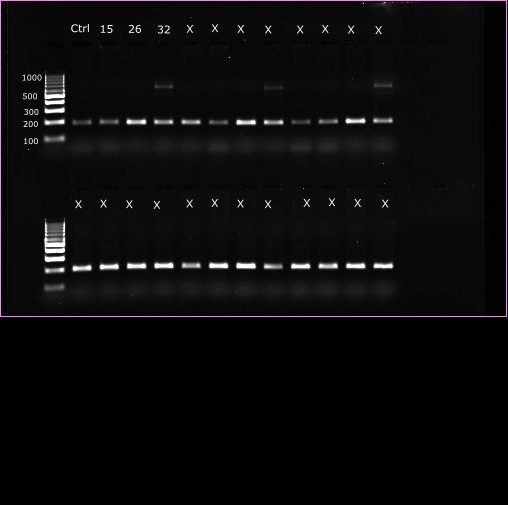


Fig 2A_raw image


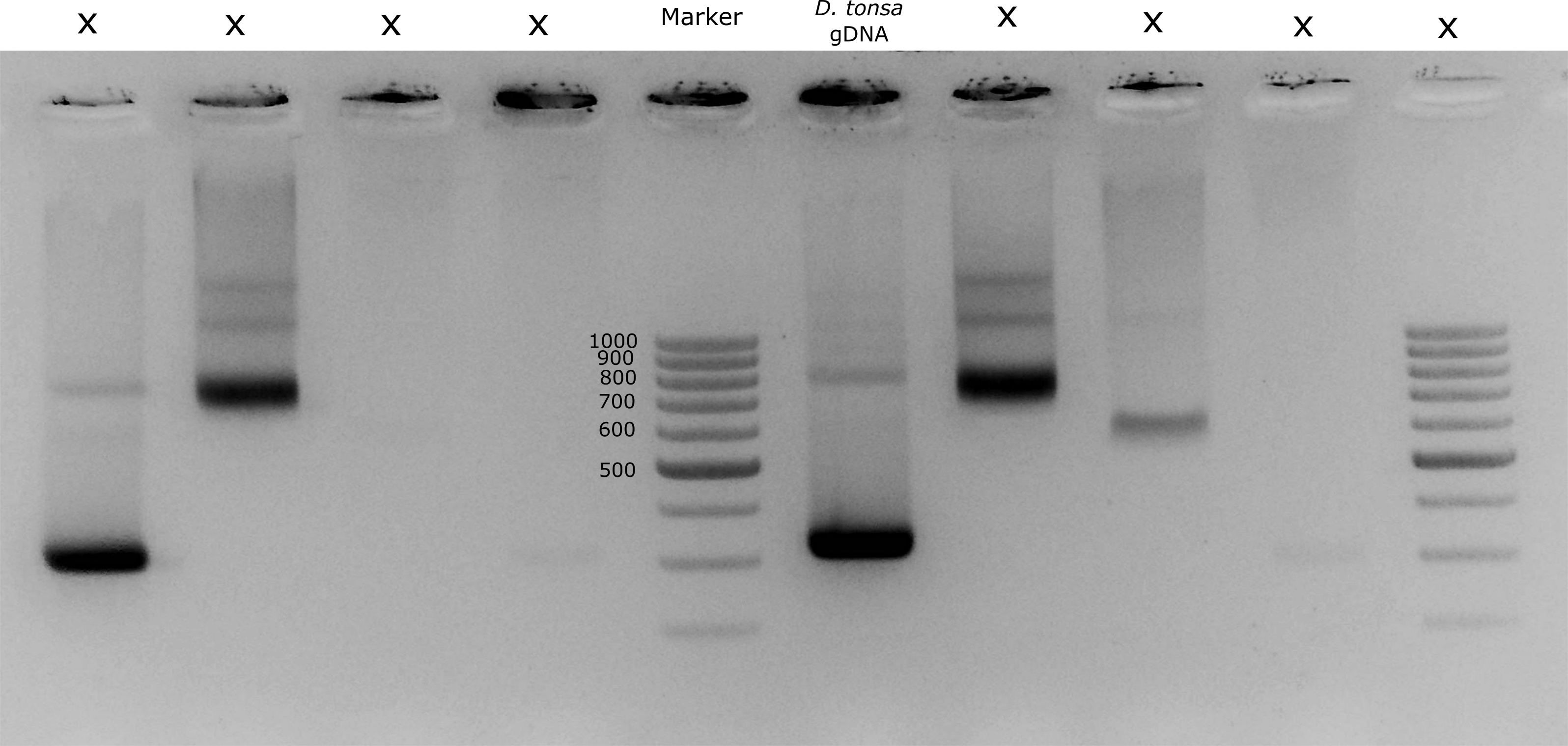


Fig 2B_raw image

Thank you for using [www.freepdfconvert.com](http://www.freepdfconvert.com/) service!

Only two pages are converted. Please Sign Up to convert all pages. <https://www.freepdfconvert.com/membership>
